# Accuracy, potential, and limitations of probabilistic record linkage in identifying deaths by gender identity and sexual orientation in the state of Rio De Janeiro, Brazil

**DOI:** 10.1186/s12889-024-19002-x

**Published:** 2024-06-01

**Authors:** Ricardo de Mattos Russo Rafael, Kleison Pereira da Silva, Helena Gonçalves de Souza Santos, Davi Gomes Depret, Jaime Alonso Caravaca-Morera, Karen Marie Lucas Breda

**Affiliations:** 1 School of Nursing, Public Health Nursing Department, State University of Rio de Janeiro, Rio de Janeiro, Brazil; 2 University of Costa Rica, San José, Costa Rica; 3 Department of Nursing, College of Education, University of Hartford, Nursing & Health Professions. West Hartford, Connecticut, United States of America

**Keywords:** Epidemiology, Gender identity, Sexual orientation, Probability, Mortality records

## Abstract

**Background:**

Globally, the counting of deaths based on gender identity and sexual orientation has been a challenge for health systems. In most cases, non-governmental organizations have dedicated themselves to this work. Despite these efforts in generating information, the scarcity of official data presents significant limitations in policy formulation and actions guided by population needs. Therefore, this manuscript aims to evaluate the accuracy, potential, and limits of probabilistic data relationships to yield information on deaths according to gender identity and sexual orientation in the State of Rio de Janeiro.

**Methods:**

This study evaluated the accuracy of the probabilistic record linkage to obtain information on deaths according to gender and sexual orientation. Data from two information systems were used from June 15, 2015 to December 31, 2020. We constructed nine probabilistic data relationship strategies and identified the performance and cutoff points of the best strategy.

**Results:**

The best data blocking strategy was established through logical blocks with the first and last names, birthdate, and mother’s name in the pairing strategy. With a population base of 80,178 records, 1556 deaths were retrieved. With an area under the curve of 0.979, this strategy presented 93.26% accuracy, 98.46% sensitivity, and 90.04% specificity for the cutoff point ≥ 17.9 of the data relationship score. The adoption of the cutoff point optimized the manual review phase, identifying 2259 (90.04%) of the 2509 false pairs and identifying 1532 (98.46%) of the 1556 true pairs.

**Conclusion:**

With the identification of possible strategies for determining probabilistic data relationships, the retrieval of information on mortality according to sexual and gender markers has become feasible. Based on information from the daily routine of health services, the formulation of public policies that consider the LGBTQ + population more closely reflects the reality experienced by these population groups.

**Supplementary Information:**

The online version contains supplementary material available at 10.1186/s12889-024-19002-x.

## Background

Mortality has been considered an important indicator in the context of public health, especially in the area of health surveillance and for public policymakers in Brazil and worldwide. It is not by chance that numerous studies address this topic [1–4]. This is because monitoring of death rates not only helps to identify the patterns and causes of death of population groups but also is useful in defining priorities for the allocation of resources and evaluating the quality of life and well-being of populations, in addition to allowing the measurement of the efficiency of the programs implemented [5, 6]. Given the importance of monitoring these data, since the 1970s Brazil has implemented one of the most robust information systems for monitoring deaths, the *Sistema de Informação sobre Mortalidade* (Mortality Information System – SIM). Under the administration of the Brazilian Ministry of Health and fully integrated into the *Sistema Único de Saúde* (Unified Health System), SIM can publish detailed data on the cause of death using the International Classification of Diseases-10 and several individual and clinical characteristics [7–9].

On the other hand, it is still not possible to say that information on deaths truly reaches all population groups. The literature is vast that points out that lesbian, gay, bisexual, *travesti*, transgender, and queer individuals (LGBTQ+; using the “plus” sign to represent the broad variety of sexual and gender identities) have worse health indicators than others group [10–15]. However, SIM still does not monitor specific information on sexual orientation and gender identity. Thus, *travestis* and trans women who were unable (or did not have the desire to) to rectify their names had their death records classified as belonging to the male population. The same occurs for trans men, who are classified as part of the female population. This is because only the sex assigned at birth and recorded in Brazilian administrative systems is identified in SIM [16, 17]. This is the paradox of the country that, according to nongovernmental agencies, murders the most LGBTQ + people worldwide [18].

Regarding the *travesti* population, a group which may be unfamiliar to parts of the international community, it is crucial to underscore that this is a demographic group with a gender identity that is unique to Brazil. *Travestis* express and embody a feminine gender presentation equal to that of cisgender women, despite being assigned a different gender at birth. The *travesti* community is notable for its long-standing struggle for rights and its resistance against frequent marginalization [15]. In recognition of the importance of gender self-identification and the diverse expressions thereof, this manuscript will use the term “*travesti*” to discuss such identities, comprehending the unique socio-cultural context of their experiences.

In the absence of specific information and due to the urgency of providing data that support specific public policies for these groups, the National Association of *Travestis* and Transgenders (ANTRA – *Associação Nacional de Travestis e Transexuais*), as well as gay activist groups, monitors deaths based on newspaper reports and information captured from hospital nets [18]. These cases, exclusively focused on murders, represent only a small part of the concrete reality experienced by these people. In other words, the country is very far from being accurate with the mortality profile of LGBTQ + populations, harming everything from the design of public policies to the direct care provided by healthcare professionals (still under the haze of the unknown due to the absence of data).

Since June 15, 2015, information on sexual orientation and gender identity in the Brazilian health information systems, has been systematically collected using only the interpersonal and self-inflicted violence module of the *Sistema de Informação de Agravos de Notificação* (Notifiable Diseases Information System - SINAN) [19]. All suspected or confirmed violent events are recorded, providing a way to expand the dataset on the LGBTQ + population. With possible inaccuracies, (due to the absence of a unique key to identify the population in information systems), the possibility of a probabilistic relationship of data may suggest a tool to estimate deaths within the Brazilian LGBTQ + population. Data relationships are a statistical tool for combining different sets of information: deterministically, when there is a unique key (identifying variable) for linking, or probabilistically when keys are used to estimate the probability that records with different names refer to the same person [20–25]. As a way of contributing to the systematic integration of the technique in health services, this study aims to evaluate the accuracy, potential, and limits of the probabilistic relationship of data to obtain information on deaths according to gender identity and sexual orientation in the State of Rio de Janeiro.

## Methods

### Study design

This manuscript stems from the data collected during the first five years of the study titled “Effects of (trans)gender identity and sexual orientation on notification and mortality due to violence: a cohort study.” Therefore, this manuscript presents a cross-sectional study that quantifies the accuracy of probabilistic data linkage to offer insights into mortality rates according to gender identity and sexual orientation. The analysis incorporates data from individuals registered in SINAN for interpersonal and self-inflicted violence, as well as data from the SIM of 92 municipalities in the state of Rio de Janeiro, Brazil, for the period from June 15, 2015, to December 31, 2020.

### Population and selection criteria

To determine the probabilistic relationships between the databases, the SINAN records and occurrence of interpersonal/self-inflicted violence between June 15, 2015 and December 31, 2020. The beginning of this period (June 15, 2015) was established based on the introduction date of collecting the variables ‘sexual orientation’ and ‘gender identity’ in the SINAN.

The records of adults aged 19 to 59 years were included. Records without information or with invalid data on birthdate, name, mother’s name, and date of occurrence of violent acts were excluded. For SIM qualification, records without identification or with invalid names or mothers’ names (e.g., indigent, male, black male, white female, etc.) were excluded. To reduce the number of records and accelerate the data relationship process, records of people younger than 19 years of age were excluded, as this was the criterion for selection in SINAN.

### Study variables

#### Linkage key variables

The variables used as link keys between the databases were “name”, “mother’s name”, and “birthdate” from the SINAN dataset. Additionally, the variables “notification number”, “date of notification”, and “date of occurrence” were sourced from SINAN, while “death number”, and “date of death” were obtained from the SIM dataset. These variables were utilized to construct an identifier code and to verify duplicate records.

### Variables of interest

The main variable under investigation was death, which was identified and retrieved from the SIM through a linkage process between databases. All deaths recorded during the study period (from June 15, 2015, to December 31, 2020), regardless of cause, were included in this analysis.

Variables such as sex, sexual orientation, and gender identity, which were of interest in this investigation, were synthesized to build analytical groups approximating the realities of exposure of people to the concrete reality. Originally present in SINAN, the possible entries included female (S0) and male (S1) for the variable “sex”; heterosexual (O0), bisexual or homosexual (O1), and “unknown” or “omitted” (O2) for “sexual orientation”; and, for the gender variable, it is important to consider that the registration was made only for “*travestis”* and “transgender women” (G1) and “*transgender* men” (G2), with the interpretation that data “unknown” or “omitted” (G0) referred to the cisgender population, despite the validity problems that this may produce [17].

**Fig. 1:**
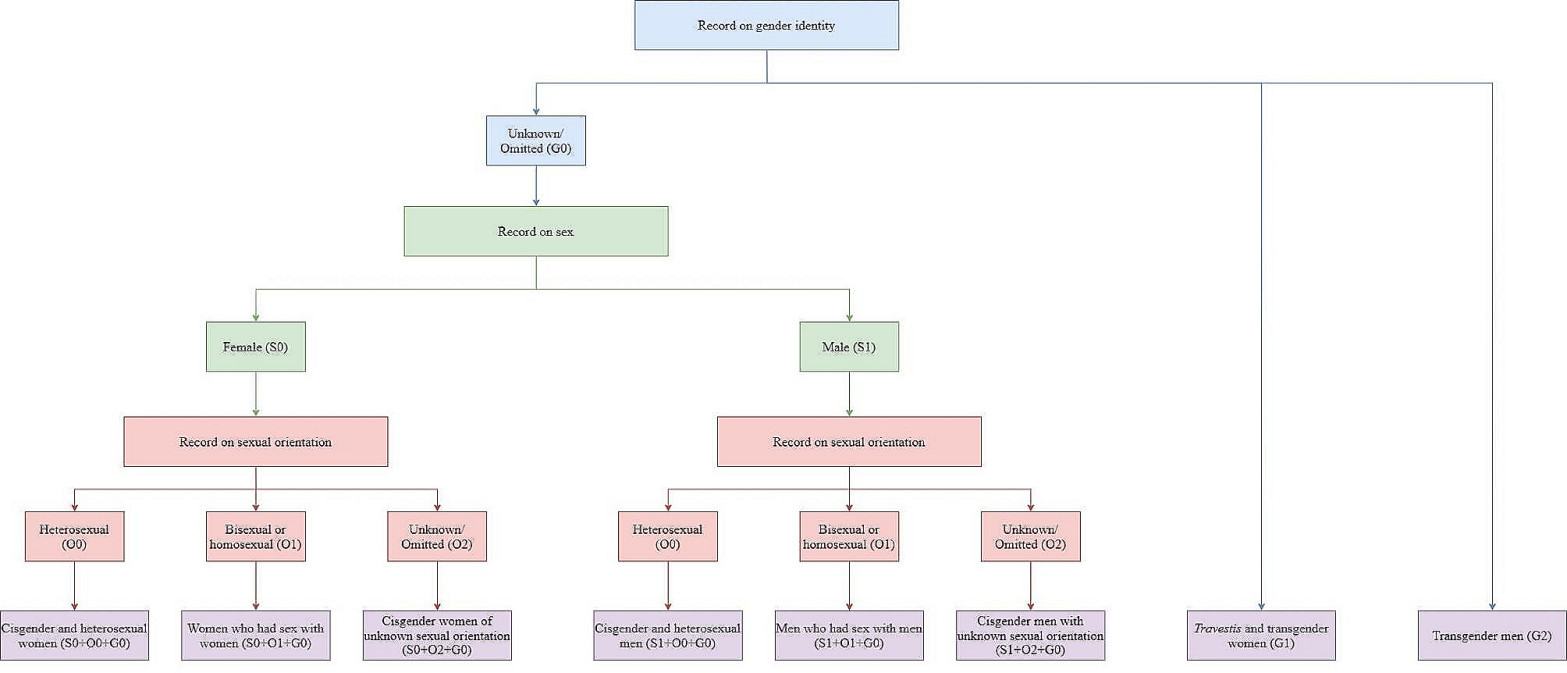
Flowchart of study’s interest groups based on sex, gender identity, and sexual orientation variables from SINAN. State of Rio de Janeiro, Brazil, 2015–2020

Thus, eight groups were created, as demonstrated by Fig. 1. The participants were cisgender and heterosexual women (S0 + O0 + G0), women who had sex with women (S0 + O1 + G0), cisgender women of unknown sexual orientation (S0 + O2 + G0), *travestis* and transgender women (G1), cisgender and heterosexual men (S1 + O0 + G0), men who had sex with men (S1 + O1 + G0), cisgender men with unknown sexual orientation (S1 + O2 + G0), and transgender men (G2). Due to the limited number of cases retrieved from the databases, the sexual orientations of transgender people were not combined in the groups formed, limiting the definition of interest groups.

### Population characteristics variables

Drawing from the SINAN database, variables were selected to characterize the investigated population. The “age group” was defined by categorizing the continuous numerical variable “age” into four adult strata. The other variables used to characterize the population included “marital status”, “schooling”, and “self-declared race/color”. The variables “person with a disability” and “people with mental or behavioral disorders” were considered as “yes” if at least one disability, and mental or behavioral disorder respectively, were documented in the database. Similarly, the “referral to the care network” was marked as “yes” if there was at least one referral to social, legal, or healthcare services documented. The “chronicity of violent episodes” variable was constructed based on the repeated entries in the SINAN database, as explained in the section on probabilistic data linkage.

### Routines of the probabilistic relationship of data

#### Stage 1: preprocessing

The first stage, titled preprocessing, was intended for the preparation and standardization of the databases for the probabilistic relationship of the data [21]. In this stage, computational scripts were built in Python 3.11.2 software in the Visual Studio Code 1.84.2 environment. Initially, the databases were reduced, leaving only the variables of interest for linking the databases (notification number/death number, date of notification, date of occurrence/date of death, name, mother’s name, and birthdate) to facilitate the process. The study selection criteria were also applied, and unique keys were constructed for each record in the database. This construction was necessary because, at least in SINAN, the notification number, which should be unique for each record, shares the same number with other people. In other words, different people had the same notification number without being homonymous. Thus, the unique key was generated based on the combination of the notification (or death) number, birthdate, first and last name, and mother’s name of each person.

With the expectation of reducing potential spelling errors in name records, a set of standardizations was implemented. This stage of standardizing letters and dates serves as a critical phase in the preprocessing for probabilistic linkage. Specifically, in the case of names, variations in spelling and the presence of special characters can increase discrepancies between records across two databases even when they pertain to the same individual. Consequently, standardizing records, particularly in personal identification, enhances the likelihood of matching between two distinct data sources [21, 23, 25].

Thus, all letters were converted to uppercase letters. Accents, special characters, excessive spaces, and other punctuation were eliminated (“Ç” became “C”, “Á” became “A”, etc.), as were prepositions (“DE”, “DE”, “DO”, “DA”, “DOS”, “DAS”, “E”). Duplicate letters were kept only once (e.g., “TT” became “T”). The letters were standardized according to Portuguese phonetics. In this context, the syllables “WA”, “KA”, “KO”, “KU”, “CE”, “CI”, “GE”, and “GI” were replaced by “VA”, “CA”, “CO”, “CU”, “SE”, “SI”, “JE”, and “JI”, respectively. The names starting with “H” had this letter deleted (e.g., “HUGO” became “UGO”), and the letter “Y” was replaced by “I”. With this approach, the standardization of syllables takes phonetics into account [21].

To handle last names in the preprocessing phase, the names were separated into fragments (first name, middle name, and last name) to optimize the next steps of building data relationships. Dates were standardized to write the day, month, and year as two, two, and four numerals, without separation by slashes.

### Stage 2: initial deduplication

The following steps, which included the completion of database preparation and the steps related to the probabilistic relationship of the data itself, were conducted in Link Plus version 3.0. This software, developed by the USA’s Centers for Disease Control and Prevention, was initially designed for the probabilistic relationship of cancer registries in the USA [26]. However, in the field of public health, its use has spread in other contexts and countries, including Brazil [22, 25, 27–29].

Thus, to finalize the preparation of the databases, the deduplication technique was applied due to the possibility of multiple records in SINAN since the recurrence of different violent events throughout life and multiple notifications of the same events are quite likely. Events may be recorded by more than one professional or health unit. The deduplication technique consists of verifying these repeated records in a database [21, 23]. Cases of duplicity were treated according to the following rules: (1) When the occurrence number was the same, with the same notification and occurrence dates, victim’s name, mother’s name, and birthdate, even allowing for spelling variations indicative of error, the record was considered the same, so only one such record was selected. (2) When the date of occurrence was different but the name, mother’s name, and birthdate were the same, even with the presence of spelling changes, it was considered a recurrence of the event, which we treated as a recurrent/chronic case. In this second rule, only the oldest record was selected, and we counted the number of repeated events in a new variable named “chronicity of violent episodes”.

### Stage 3: blocking and matching

After standardization and preprocessing were completed, the blocking and pairing stage began. Blocking is a method of creating logical subsets based on specific criteria of variables to reduce the number of comparisons during the database relationship. This reduces the processing time. Pairing is intended to compare the corresponding records in the databases using preestablished algorithms in the relationship software [21, 23].

In the specific case of this study, nine blocking and pairing strategies were compared. The blocking strategies combined the first name (FN), last name (LN), mother’s first name (FM), mother’s last name (LM), and birthdate (BD) using the soundex code to reduce potential spelling errors and nominal variations. For the pairing strategies, combinations of the full name (N), mother’s name (M), and birthdate (BD) were used, defining the minimum probabilities of agreement (M-probability) as 0.95 and 0.65 for the name and birthdate, respectively. In addition, the direct method of pairing and an initial cutoff point of 10 were used for the scores calculated from the data.

### Stage 4: manual review of pairs

A manual peer review was performed for each strategy. The defining criteria for true pairs were as follows: (1) same name, birthdate, and mother’s name; (2) same name, mother’s name, and day and month of birth but with a variation of ± 2 years for the last digit of the year of birth; (3) same name, mother’s name, and year of birth, with inversion of the day and month digits; (4) same name, mother’s name, and day and year of birth; (5) same name, mother’s name, and month and year of birth; (6) similar name and mother’s name with variation in the orthographic scan; and (7) unusual name and mother’s name (foreign names, for example) that agree on the date of birth.

### Stage 5: Postpairing

In the postpairing phase, a new deduplication was applied to the sets of pairs formed in each strategy. The selection of the best matching and blocking strategy was based on the shortest processing time and the most true pairs after deduplication. Once the best strategy was found, the files were combined based on the unique key constructed during the preparation phase, thus joining the true pairs (deaths) with the SINAN database and all its variables. The same occurred with the SIM variables, specifically concerning true pairs. The bank combinations were performed using Stata SE 15 software.

### Statistical analysis

We analyzed the processing times, minimum and maximum scores, absolute numbers, and proportions of true pairs formed by each blocking and pairing strategy in the probabilistic data relationship. We determined the criteria that composed the optimal strategy. We computed the areas under the receiver operating characteristic (ROC) curves to determine the best cutoff point for the matching scores. In addition, the sensitivity (%), specificity (%), accuracy (%), positive and negative likelihood ratios, number of true pairs, and number of false pairs were calculated for each cutoff point of the scores of the optimal strategy.

After integrating the SINAN database with the true pairs data from the SIM, and determining the optimal matching strategy, we calculated the proportions for each variable of interest in the study. This process also involved determining the number of deaths from any cause within this specific group and calculating the mortality rates per thousand individuals. These rates were derived by comparing the total number of deaths identified through the linkage of the SIM and SINAN databases against the records of violence victims listed in SINAN. This approach has allowed us to gauge the mortality among violence victims recorded in SINAN, without intending to establish a direct causality between the violent incidents occurring over time and death.

We then analyzed these rates to identify differences between the initial cut-off point and the one chosen through the most effective matching strategy. In addition, with the objective of observing the existence of differences between the cutoff points, the proportion of agreement, the kappa statistic, and the respective p values were calculated for each covariate of the study. The interpretation of the kappa statistic was classified as full agreement (0.81-1.00), substantial agreement (0.61–0.80), moderate agreement (0.41–0.60), fair agreement (0.21–0.40), slight agreement (0-0.20), or no agreement (< 0) [30, 31]. All analyses were performed using Stata SE 15 software.

### Ethical considerations

Our study was conducted following Brazilian ethical guidelines for research involving human subjects. The State University of Rio de Janeiro Research Ethics Committee (Rio de Janeiro, Brazil) waived the need for informed consent, and reviewed and approved the study under protocol number 5,009,244.

## Results

### Data preparation and matching strategy

**Fig. 2:**
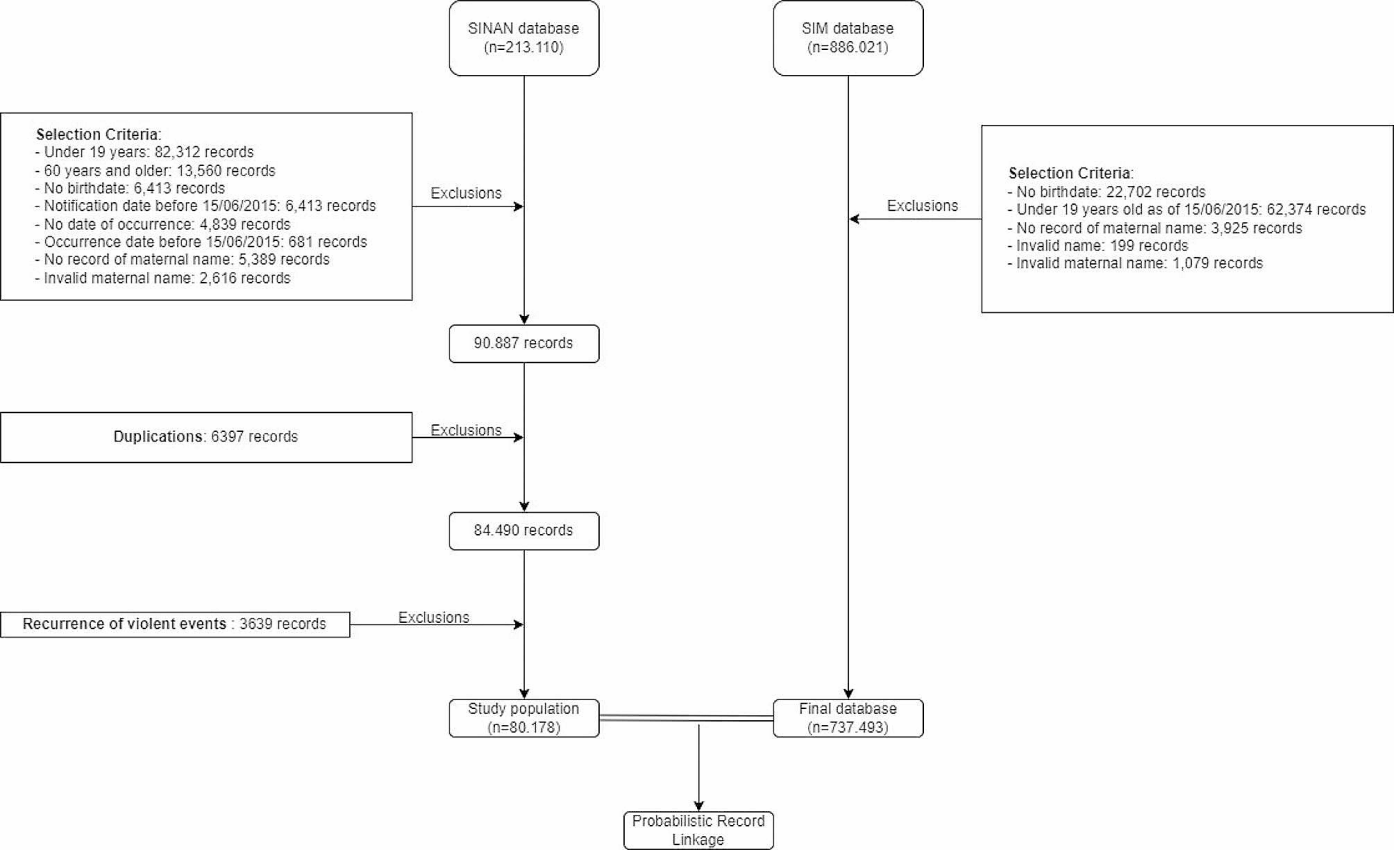
Flowchart of the study population composition for probabilistic record linkage. State of Rio de Janeiro, Brazil, 2015–2020

Figure 2 shows the sample composition that formed the baseline of the SINAN probabilistic relationship of interpersonal and self-inflicted violence, as well as the SIM rating. Note that 60.35% (*n* = 128,620) of the records were excluded from SINAN based on the selection criteria. In addition, 3639 cases of notification recurrence were identified—i.e., when the case was the same but with different events and dates. In this case, only one record was kept, for which purpose we created a specific variable, which represented the chronicity of violent episodes. Thus, the baseline consisted of 80,178 records. In turn, SIM had 90,279 (10.12%) exclusions of records based on the qualification criteria of the database, totaling 737,493 death records (Fig. 1).

Table 1 presents the results of the probabilistic relationship according to the 9 blocking and pairing strategies. strategy 1, although not the one with the highest percentage of true positives after deduplication, had the highest absolute number of true pairs (*n* = 1156) and the shortest data processing time (11 min and 50 s). Therefore, according to the preestablished criteria, the best strategy for further analysis was strategy 1.

**Table 1:** The results of probabilistic record linkage according to blocking and matching strategies. State of Rio de Janeiro, Brazil, 2015–2020

ID	Blocking strategy	Matching strategy	Score	Processing Time	P1	TP1	%TP1	PD	P2	TP2	%TP2
1	FN + LM	N + BD + M	10.6–29.7	00:11:50	4087	1578	38.61	22	4065	1556	38.28
2	FN + LN + BD + FM + LM	N + BD + M	10.6–29.7	02:28:54	7296	1570	21.52	19	7277	1551	21.31
3	FN + LN + FM + LM	N + BD + M	10.6–29.7	00:25:46	7262	1570	21.62	20	7242	1550	21.4
4	FN + LN + FM + LM	N + BD	12.1–20.0	00:20:24	2388	1111	46.52	10	2378	1101	46.3
5	FN + LN + FM + LM	BD + M	10.6–18.5	00:19:11	5561	996	17.91	9	5552	987	17.78
6	FN + LN + BD	N + BD + M	10.6–29.7	02:19:07	7296	1570	21.52	20	7276	1550	21.3
7	FN + LN + BD	N + BD	12.1–20.0	01:32:52	2397	1103	46.02	10	2387	1093	45.79
8	FN + BD	N + BD + M	10.6–29.7	02:10:48	7296	1570	21.52	20	7276	1550	21.3
9	BD	N + BD + M	10.6–29.7	02:07:21	7296	1570	21.52	20	7276	1550	21.3

Legend

ID: identification of the data linkage strategy; P1: pairs formed after data linkage; TP1: proportion of true pairs formed after data linkage; DP: number of duplicate pairs; P2: pairs formed after the deduplication strategy; TP2: true pairs formed after deduplication; %TP2: proportion of true pairs formed after deduplication; DB: birthdate; N: name; FN: soundex of the first name; LN: soundex of the last surname; M: mother’s name; FM: soundex of the mother’s first name; LM: soundex of the mother’s last surname

### Performance of the best matching strategy

**Fig. 3:**
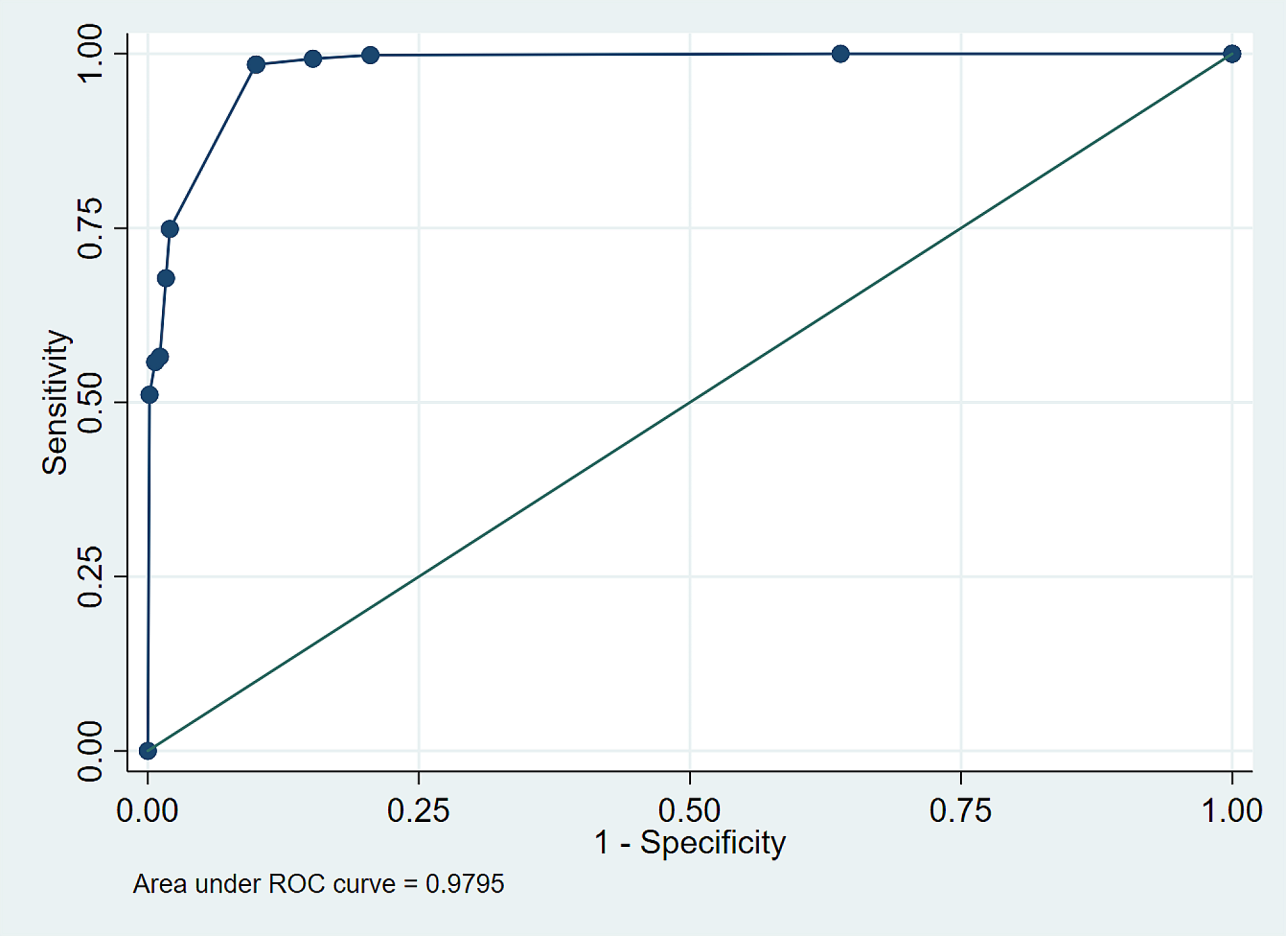
ROC curve of the best blocking and matching strategy (strategy number 1). State of Rio de Janeiro, Brazil, 2015–2020 (*n* = 4065)

Figure 3 shows the ROC curve of strategy 1. With an area under the curve of 0.979 (95% confidence interval: 0.976–0.983), the probabilistic relationship has an excellent ability to discriminate between true and false pairs. Table 2 lists the properties of the strategy. When a cutoff point of 17.9 was used for the score, the sensitivity was 98.46%, the specificity was 90.04%, and the positive and negative likelihood ratios were 9.84 and 0.02, respectively. This means this cutoff point allowed the correct identification of 98.46% of the true pairs and 93.26% of the false pairs. In addition, the likelihood ratio values show that a positive rating in the relationship significantly increased the chances of the pair being truly positive, while a negative classification, due to the very low negative likelihood ratio, provided strong evidence that the pair was not a true pair. Finally, when analyzing the accuracy, we observed that this cutoff point could correctly classify 93.26% of the observations, i.e., 1532 (98.46%) of the 1556 true pairs, as well as correctly classify 2259 (90.04%) of the 2509 false pairs, greatly reducing the need for manual review after the probabilistic relationship of the data.

**Table 2:** Accuracy by cutoff point of the best blocking and matching strategy (strategy 1). State of Rio de Janeiro, Brazil, 2015–2020 (*n* = 4065)

Cutoff	S%	E%	Ac%	LR+	LR-	PT	PF
≥ 10.60	100.00	-	38.28	-	-	0	906
≥ 12.10	100.00	36.11	60.57	1.00	-	3	1.088
≥ 12.30	99.81	79.47	87.26	1.56	-	8	133
≥ 13.80	99.29	84.77	90.33	4.86	0.002	13	131
≥ 16.50	98.46	90.00	93.23	6.52	0.008	0	1
**≥ 17.90**	**98.46**	**90.04**	**93.26**	**9.84**	**0.02**	**367**	**199**
≥ 18.50	74.87	97.97	89.13	9.88	0.02	110	9
≥ 20.00	67.8	98.33	86.64	36.83	0.26	175	14
≥ 21.70	56.56	98.88	82.68	40.50	0.33	12	11
≥ 23.50	55.78	99.32	82.66	50.68	0.44	73	13
≥ 29.70	51.09	99.84	81.18	82.33	0.44	795	4
> 29.70	0	100.00	61.72	320.48	0.49	-	-

Legend: S%: sensitivity value expressed as a proportion; E%: specificity value expressed as a proportion; Ac%: accuracy value expressed as a proportion; LR+: positive likelihood ratio; LR-: negative likelihood ratio; TP: true pairs; FP: false pairs

### Characteristics of population, mortality rates, and linkage cutoff agreement

Table 3 presents the characteristics of the study population; the crude mortality rates for the two cutoff points of the scores generated in the probabilistic relationship of the data; and the kappa statistic for each population subgroup. The study population was mostly composed of women (76.72%), whether cisgender or transgender, aged up to 39 years (73.74%), black (65.46%), single (45.56%), and having more than 8 years of school (66.23%). A total of 2.31% of people with disabilities were identified, 4.77% of the population reported some mental or behavioral disorders, and 3.77% had chronic episodes of violence. Most patients (78.12%) had been referred to the assistance network. Compared with women, mortality rates were ~ 3 times greater in men. Notably, after excluding groups of women with an unknown sexual orientation, the women who had sex with women and *travestis* and transgender women had higher mortality rates than did cisgender women. Higher mortality rates are also observed with advancing age; in black, married and nonsingle people; with less schooling; in people with disabilities or mental and behavioral disorders; and in cases of chronic violence. In contrast, people who received referrals to the health care network had lower mortality rates. With the kappa statistic indicating full agreement for all covariates (*p* < 0.001), the adoption of the cutoff point of ≥ 17.90 did not produce substantial differences in the interpretation of the results.

**Table 3:** Characteristics of the study population, mortality rates (per 1000 persons), and agreement among the cutoff points of the best strategy (#1) for each population subgroup. State of Rio de Janeiro, Brazil, 2015–2020 (*n* = 80.178)

Variables	*N*	%	Cutoff ≥ 10.60		Cutoff ≥ 17.90		% Agreement	Kappa	*p* value
			Deaths	MR	Deaths	MR			
**Overall**	**80,178**	**-**	1556	19.41	1532	19.11	99.97	0.992	**< 0.001**
**Groups**	**80,169**	**-**	**-**		**-**		**-**	**-**	**-**
HCW	28,987	36.20	285	9.83	281	9.69	99.99	0.993	**< 0.001**
WSW	1159	1.45	13	11.22	13	11.22	100.00	1.000	**< 0.001**
CWUnknow	30,884	38.50	474	15.35	467	15.12	99.98	0.992	**< 0.001**
TGW	472	0.59	7	14.83	7	14.83	100.00	1.000	**< 0.001**
HCM	4496	5.61	172	38.26	171	38.03	99.98	0.997	**< 0.001**
MSM	534	0.67	11	20.60	10	18.73	99.81	0.951	**< 0.001**
CMUnknow	13,550	16.90	591	43.62	580	42.80	99.92	0.990	**< 0.001**
TGM	87	0.11	3	34.48	3	34.48	100.00	1.000	**< 0.001**
**Age group**	**80,178**	**-**	**-**	**-**	**-**	**-**	**-**		
19–29 years	35,327	44.06	494	13.98	483	13.67	99.97	0.989	**< 0.001**
30–39 years	23,800	29.68	360	15.13	353	14.83	99.97	0.990	**< 0.001**
40–49 years	13,848	17.27	342	24.70	339	24.48	99.98	0.996	**< 0.001**
≥ 50 years	7203	8.98	360	49.98	357	49.56	99.96	0.996	**< 0.001**
**Self-declared race/color**	**62,620**	**-**	**-**	-	-	-	**-**	**-**	**-**
White	21,001	33.54	378	18.00	371	17.66	96.50	0.990	< 0.001
Black (Black/*Pardos*)	40.991	65.46	818	19.96	808	19.71	96.98	0.994	< 0.001
Asian	495	0.79	4	8.08	4	8.08	100.00	1.000	< 0.001
Indigenous	133	0.21	2	17.70	2	15.03	100.00	1.000	< 0.001
**Marital status**	**47,110**	**-**					**-**	**-**	**-**
Married and other	21,461	45.56	347	16.17	340	15.84	99.97	0.990	**< 0.001**
Single	25,649	54.44	404	15.75	399	15.56	99.98	0.994	**< 0.001**
**Schooling**	**29,326**						-		
≥ 8 years of study	19,423	66.23	195	10.04	192	9.89	99.98	0.999	**< 0.001**
Up to 8 years of study	9903	33.77	235	23.73	231	23.33	99.96	0.991	**< 0.001**
**Persons with a disability**	**80,178**	**-**					**-**	**-**	**-**
No	78,325	97.69	1463	18.68	1440	18.38	99.97	0.992	**< 0.001**
Yes	1853	2.31	93	50.19	92	49.65	99.95	0.994	**< 0.001**
**Persons with a mental or behavioral disorder**	**80,178**	**-**					**-**	**-**	**-**
No	76,350	95.23	1368	17.92	1346	17.63	99.97	0.992	**< 0.001**
Yes	3828	4.77	188	49.11	186	48.59	99.95	0.994	**< 0.001**
**Chronicity of violent episodes**	**80,178**	**-**					**-**	**-**	**-**
No	77,159	96.23	1493	19.35	1469	19.04	99.97	0.992	**< 0.001**
Yes	3019	3.77	63	20.87	63	20.87	100.00	1.000	**< 0.001**
**Referral to the assistance network**	**80,178**	**-**					**-**	**-**	**-**
No	17,545	21.88	438	24.96	428	24.39	99.94	0.988	**< 0.001**
Yes	62,633	78.12	1118	17.85	1104	17.63	99.98	0.994	**< 0.001**

Legend: MR: Mortality Ratio; HCW - Heterosexual cisgender women; WSW - Woman who had sex with women; CWUnknow - Cisgender women of unknown sexual orientation; TGW - *Travestis* and Transgender women; HCM - Heterosexual cisgender men; MSM - Men who had sex with men; CMUnknow - Cisgender men of unknown sexual orientation; TGM - Transgender men

## Discussion

The main results of this study are focused on demonstrating the feasibility and good accuracy of determining the probabilistic relationship of data between the SINAN database of interpersonal and self-inflicted violence and the SIM database to obtain information on sexual orientation and gender identity. Keeping a certain pioneering spirit, our findings make a significant contribution to addressing critical gaps on this subject both in Brazil and globally. As noted, the utilization of data linkage techniques allows for the retrieval of information about gender and sexuality markers, a facet still relatively underexplored in research. Determining a linkage strategy, with parsimonious parameters for identifying true and false pairs, tends to contribute to the use of the technique in the daily routine of health services and in academic production.

By adopting preprocessing routines and considering the relationship time as a function of the number of true pairs detected, the recognition of the best blocking and processing strategies constitutes an advance for the systematic adoption of this method in the daily life of the services, especially when considering sexual orientation and gender identity as exposure variables in future analyses. These procedures resulted in a true-pair detection rate with proportions compatible with those found in other investigations whose outcome variable was the registration of death in SIM [20, 32].

On the other hand, it is important to note that we are faced with slightly different connecting keys between these investigations. In this article, the linking keys were restricted to the variable’s “name”, “birthdate”, and “mother’s name”. Although the municipality of residence is traditionally used in probabilistic relationships, in the specific case of our study, this variable could bring more elements of uncertainty than solutions. This is because when reaching groups such as *travestis* and transgender women, as well as people known to be victims of violence, depending on the population base used, it is imperative to consider the possibility of constant changes in addresses over time due to stigma and the structure of violence itself that surrounds them [33]. There has been a consensus in Brazilian production that housing instability is a factor present in the lives of a significant portion of *travestis* and transgender women [10, 34]. These distinctions in the probabilistic data matching strategies necessitate caution when comparing the data linkage results of different studies.

Complementing this analysis, the definition of a cutoff point in the scores of the probabilistic relationship of data constitutes another important aspect for the advancement of the adoption of this technique in analyses both by health surveillance services and by the academic sector. Recognizing the cutoff point, the manual peer review phase tends to be more efficient [4, 24], significantly reducing processing time and improving accuracy in future data relationships. The adoption of scores ≥ 17.9 reduced 56.16% of the pairs to be reviewed, with 90.04% of the pairs being truly negative. In other words, with high specificity (> 90.00%) and with the loss of only 25 true pairs erroneously classified as false (98.46% sensitivity), our strategy 1 demonstrated excellent properties and elements compatible with other linkage strategies with Brazilian datasets [20, 32]. In addition, when incorporating the deaths into the SINAN database, the degrees of agreement for the kappa indicator are high and close to 1 for all the variables of interest in this study, revealing that the adoption of a higher cutoff point (≥ 17.9) does not imply losses for future analyses.

The mortality rates observed at the two cutoff points established by the linkage strategy revealed a scenario consistent with the investigations on the subject. That is, advanced age, black race/color, a shorter length of schooling, and disabilities and mental and behavioral disorders [35–37] were associated with higher mortality rates. Thus, these results corroborate the effectiveness of the probabilistic data relationship strategy in the production of reliable information on this topic.

The same occurs in relation to gender markers and sexual orientation. It was observed that transgender women and those who have sex with other women have higher mortality rates than heterosexual cisgender women. Men, whether they are cisgender or transgender, as well as men who have sex with men, also have a higher risk of dying than heterosexual cisgender women [38–41]. Thus, having established the cutoff point and the best strategies for capturing information in large datasets, it is essential to scrutinize in more detail how sexual and gender markers affect the risk of dying in the population.

Furthermore, the number of deaths among *travestis* and transgender people is likely higher than that captured in the linkage process, resulting in classification bias. While cisgender men and women have their names established passively, that is, during birth [42], transgender and nonbinary people are actively involved in the choice of this name and still face important bureaucratic barriers to be able to make it compatible with their identity [16]. It’s conceivable that the name recorded in the SINAN form for some transgender individuals may have been changed over time [19]. It’s plausible to consider that the deaths of some transgender individuals may not have been accurately captured during the linkage process. In these cases, the death will not be attributed to the person, resulting in a false-negative result and, once again, approaching the mortality rates between cisgender and transgender people.

Additionally, although the use of the method is promising for the analysis of mortality according to sexual orientation and gender identity, other precautions are necessary. Although the quality of filling out the SINAN has improved over the years [43], notably, the present study found that sexual orientation was not reported (i.e., it was considered “unknown” or “omitted”) in 44.45% of the patients. A similar situation can be observed regarding “self-reported race/color”, “marital status”, and, even more markedly, regarding the variable “schooling”.

The quality and completeness of information in Brazilian health information systems have been constant and long-standing challenges for researchers and health managers since the omission of such data greatly compromises the analytical capacity and formulation of public policies [44–46]. Because they are constant variables and are known to be associated with asymmetries and inequities in health [47–51], their omission in analytical models, either as a confounding variable or, in some cases, as an exposure variable, can produce strong biases in the production of knowledge on the subject. In this vein, reflecting on professional awareness strategies for the incorporation of these practices of sensitive and attentive questioning to users, as well as awareness that these factors are preponderant for the construction of health actions, are currently urgent elements.

The classification of the “gender identity” variable in SINAN demands careful attention as the system prescribes specific categories for *travestis*, transgender women, and transgender men [19]. Consequently, individuals who do not fit these specified categories or whose identities were not recorded — labeled in the system as “unknown” or “omitted” — are automatically classified as cisgender. This oversimplified approach might not accurately capture the full spectrum of gender identities, particularly for those whose true identity was either not recorded or not inquired about. This limitation can compromise the tool’s sensitivity and, in turn, in mortality analyses, data about the cisgender population may be skewed by inaccurately categorized deaths. This leads to a potentially flawed estimation of mortality rates among cisgender individuals compared to the groups of *travestis*, transgender women, and transgender men [17].

In addition to the limits already exposed and which must be considered when interpreting probabilistic relationships with these datasets, an important selection bias must be considered in future analyses. When studying one of the few databases that contains information on sexual orientation and gender identity, the SINAN database of interpersonal and self-inflicted violence, we partially apprehended the actual information experienced by the population. This is because not all people suffer violence, and not all people who suffer violence are notified in health services.

Studies have shown substantial differences in the prevalence of violence between primary surveys and the results obtained by SINAN [52–54]. A notable example of these differences is the fact that the surveys indicate psychological violence as the most prevalent [55], while the SINAN data indicate physical violence as the one with the greatest magnitude of violent events [43]. This difference may be associated with health professionals’ own understanding of what the phenomenon of violence is, as well as the interpretation of which violence is worthy of reporting and which is not, since notions about this phenomenon are influenced by the sociohistorical conformations of society [56]. In other words, culture, values, and social structure directly affect people’s perceptions of what is and is not considered violence. They also produce a kind of stratification of which types of violence are more violent than others and, consequently, those that are worthy of immediate reporting and those that do not need to be recorded urgently [57]. Thus, physical and sexual violence, as well as cases that imminently threaten life, might be more common than others.

This information bias results in the generation of statistics based on SINAN data that differ from the reality estimated by surveys [43, 52–55]. In this context, analyses derived from linkages whose population base is based on SINAN should be interpreted with caution, as the records refer to a population subjected to specific contexts that may be different from those observed in the general population. Thus, the mortality rates observed in our study should not be taken as representative of the general population. On the contrary, their utility lies in the ability to conduct repeated measures over time, providing the monitoring of mortality according to sexual orientation and gender identity. As mentioned, the SINAN is the only Brazilian database where sexual and gender markers are officially monitored.

This bias can be circumvented by introducing a unique identification key [16] in information systems, such as civil registry numbers, the natural persons registry, or the national registry of the health system, as well as the introduction of the variables “sexual orientation” and “gender identity” into other data collection systems and not only in the SINAN database of interpersonal and self-inflicted violence [16, 58]. Until then, even with recognized inaccuracies and analytical limits, the performance of procedures involving probabilistic data relationships should be encouraged to better understand the needs of certain subpopulations, improve the technique for capturing information about the LGBTQ + population, and thereby advance the formulation of public policies compatible with the needs of this group.

## Conclusion

Despite the inaccuracies produced by the data collection format of the variables “gender identity” and “sexual orientation”, the probabilistic data linkage is an important technique with high applicability in the daily routine of health services. With its identification of possible blocking and pairing strategies and the detection of the best cutoff point for linkage scores, this technique becomes more useful in daily research and monitoring by health surveillance services. The application of this technique should be considered in monitoring mortalities over time, as it is useful in formulating public policies for the LGBTQ + population.

### Electronic supplementary material

Below is the link to the electronic supplementary material.

Supplementary Material 1

## Data Availability

For ethical reasons, we cannot provide the identified data necessary for reproducing the probabilistic record linkage. The anonymized data after the probabilistic record linkage are available as supplementary material.

## Abbreviations

Ac%: Accuracy value expressed as a proportion
ANTRA: Brazilian National Association of Travestis and Transgenders
BD: Birthdate
CMUnknow: Cisgender men of unknown sexual orientation
CWUnknow: Cisgender women of unknown sexual orientation
E%: Specificity value expressed as a proportion
FM: Mother’s first name
FN: First name
FP: False pairs
HCM: Heterosexual cisgender men
HCW: Heterosexual cisgender women
LGBTQ+: Lesbian, gay, bisexual, travesti, transgender, and queer individuals, as well as people who identify with other gender identities and sexual orientations that are not heterosexual and cisgender
LM: Mother’s last name
LN: Last name
LR-: Negative likelihood ratio
LR+: Positive likelihood ratio
M: Mother’s name
MR: Mortality ratio
MSM: Men who had sex with men
N: Full name
ROC: Receiver operating characteristic
S%: sensitivity value expressed as a proportion
SIM: Mortality information system
SINAN: Notifiable Diseases Information System
TGM: Transgender men
TGW: Travestis and Transgender women
TP: True pairs
WSW: Woman who had sex with women

## References

[1] Baqui P, Bica I, Marra V, Ercole A, van der Schaar M (2020). Ethnic and regional variations in hospital mortality from COVID-19 in Brazil: a cross-sectional observational study. Lancet Glob Health.

[2] Ramos D, da Silva NB, Ichihara MY, Fiaccone RL, Almeida D, Sena S (2021). Conditional cash transfer program and child mortality: a cross-sectional analysis nested within the 100 million Brazilian cohort. PLoS Med.

[3] Bollyky TJ, Templin T, Cohen M, Schoder D, Dieleman JL, Wigley S (2019). The relationships between democratic experience, adult health, and cause-specific mortality in 170 countries between 1980 and 2016: an observational analysis. Lancet.

[4] Rebora P, Scirè CA, Occhino G, Bortolan F, Leoni O, Cideni F et al. Development and validation of an electronic database-based frailty index to predict mortality and hospitalization in a population-based study of adults with SARS-CoV-2. Front Med (Lausanne). 2023;10. 10.3389/fmed.2023.1134377 PMC10213394 37250632

[5] Phyo AZZ, Freak-Poli R, Craig H, Gasevic D, Stocks NP, Gonzalez-Chica DA (2020). Quality of life and mortality in the general population: a systematic review and meta-analysis. BMC Public Health.

[6] Batty GD, Kivimäki M, Frank P (2022). State care in childhood and adult mortality: a systematic review and meta-analysis of prospective cohort studies. Lancet Public Health.

[7] Ministério da Saúde (2022). Declaração De Óbito: manual de instruções para preenchimento.

[8] de Morais RM, Costa AL. Uma avaliação do Sistema de Informações sobre Mortalidade. Saúde em Debate. 2017;41 spe:101–17.

[9] Ranzani OT, Marinho M, de Bierrenbach F. AL. Utilidade do Sistema De Informação Hospitalar na vigilância da mortalidade materna no Brasil. Revista Brasileira De Epidemiologia. 2023;26. 10.1590/1980-549720230007.2 PMC9838231 36629619

[10] Rafael R, de MR, Jalil EM, Luz PM, de Castro CRV, Wilson EC, Monteiro L (2021). Prevalence and factors associated with suicidal behavior among trans women in Rio De Janeiro, Brazil. PLoS ONE.

[11] Montenegro L, Velasque L, LeGrand S, Whetten K, Rafael R, de MR, Malta M (2020). Public Health, HIV Care and Prevention, Human Rights and Democracy at a crossroad in Brazil. AIDS Behav.

[12] Spittlehouse JK, Boden JM, Horwood LJ (2020). Sexual orientation and mental health over the life course in a birth cohort. Psychol Med.

[13] Semlyen J, King M, Varney J, Hagger-Johnson G (2016). Sexual orientation and symptoms of common mental disorder or low wellbeing: combined meta-analysis of 12 UK Population health surveys. BMC Psychiatry.

[14] Bränström R, Narusyte J, Svedberg P (2023). Sexual-orientation differences in risk of health-related impaired ability to work and to remain in the paid workforce: a prospective population-based twin study. BMC Public Health.

[15] Breda KML, Carava-Morera JA, de Rafael R, de Chesnay M, Sabella D (2023). Trans trafficking and sex work in Brazil, Costa Rica, and the USA. Human trafficking: A Global Health Emergency.

[16] Rafael R, de MR, Santos HG, de Caravaca-Morera S, Wilson JA, Breda EC. KL. Inclusão Ou ilusão Da Identidade De gênero no país com o maior número de assassinatos de transgêneros: um ensaio crítico brasileiro. Escola Anna Nery. 2023;27.

[17] Rafael R, de MR, Gil AC, Santos HG, de Caravaca-Morera S, Breda JA. KL. Ensaio teórico-metodológico sobre validade da informação da identidade de gênero no monitoramento epidemiológico da violência. Volume 57. Revista da Escola de Enfermagem da USP; 2023.

[18] Benevides BG, Nogueira SNB (2020). Dossiê dos Assassinatos E Da violência contra travestis e transexuais no Brasil em 2018.

[19] Ministério da Saúde. Viva: instrutivo notificação de violência interpessoal e autoprovocada. 2nd edition. Brasília: Ministério da Saúde; 2016.

[20] de Oliveira GP, Bierrenbach AL, de Camargo Júnior S, de Coeli KR, Pinheiro CM. RS. Accuracy of probabilistic and deterministic record linkage: the case of tuberculosis. Rev Saude Publica. 2016;50. 10.1590/S1518-8787.2016050006327 PMC4988803 27556963

[21] Machado JP, da Silveira DP, Santos IS, Piovesan MF, Albuquerque C (2008). Aplicação Da metodologia de relacionamento probabilístico de base de dados para a identificação de óbitos em estudos epidemiológicos. Revista Brasileira De Epidemiologia.

[22] Quezada-Sánchez AD, Espín-Arellano I, Morales-Carmona E, Molina-Vélez D, Palacio-Mejía LS, González-González EL (2022). Implementation and validation of a probabilistic linkage method for population databases without identification variables. Heliyon.

[23] Asher J, Resnick D, Brite J, Brackbill R, Cone J (2020). An introduction to probabilistic record linkage with a focus on Linkage Processing for WTC Registries. Int J Environ Res Public Health.

[24] Peres SV, Latorre M do RD, de O, Michels FAS, Tanaka LF, Coeli CM, Almeida MF. de. Determinação de um ponto de corte para a identificação de pares verdadeiros pelo método probabilístico de linkage de base de dados. Cad Saude Colet. 2014;22:428–36.

[25] Prindle J, Suthar H, Putnam-Hornstein E (2023). An open-source probabilistic record linkage process for records with family-level information: Simulation study and applied analysis. PLoS ONE.

[26] Centers for Disease Control and Prevention (CDC). National Program of Cancer Registries. Link Plus 3.0 version. 2007.

[27] Silva DRMe, Luizaga CT, de Toporcov M, Algranti TN. E. Concordância E Validade dos diagnósticos de cânceres associados ao asbesto no sistema de informação hospitalar do Sistema Único De Saúde. Revista Brasileira De Epidemiologia. 2021;24. 10.1590/1980-549720210044 34406206

[28] Agranonik M, Jung RO (2019). Qualidade dos sistemas de informações sobre nascidos vivos e sobre mortalidade no Rio Grande do sul, Brasil, 2000 a 2014. Cien Saude Colet.

[29] Ministério da Saúde (2021). Saúde Brasil 2020/2021: uma análise da situação de saúde e da qualidade da informação.

[30] Landis JR, Koch GG (1977). The measurement of Observer Agreement for Categorical Data. Biometrics.

[31] Altman DG. Practical Statistics for Medical Research. Chapman and Hall/CRC; 1990.

[32] Coeli CM, Saraceni V, Medeiros PM, da Silva Santos HP, Guillen LCT, Alves LGSB (2021). Record linkage under suboptimal conditions for data-intensive evaluation of primary care in Rio De Janeiro, Brazil. BMC Med Inf Decis Mak.

[33] Veroneze RT (2022). Vulnerabilidades das travestis e das mulheres trans no contexto pandêmico. Revista Katálysis.

[34] Grinsztejn B, Jalil EM, Monteiro L, Velasque L, Moreira RI, Garcia ACF (2017). Unveiling of HIV dynamics among transgender women: a respondent-driven sampling study in Rio De Janeiro, Brazil. Lancet HIV.

[35] Sasson I, Hayward MD (2019). Association between Educational Attainment and Causes of Death among White and Black US Adults, 2010–2017. JAMA.

[36] Momen NC, Plana-Ripoll O, Agerbo E, Christensen MK, Iburg KM, Laursen TM (2022). Mortality Associated with Mental disorders and Comorbid General Medical conditions. JAMA Psychiatry.

[37] Ferdows NB, Aranda MP, Baldwin JA, Baghban Ferdows S, Ahluwalia JS, Kumar A (2020). Assessment of racial disparities in Mortality Rates among older adults living in US Rural vs Urban counties from 1968 to 2016. JAMA Netw Open.

[38] Coelho LE, Torres TS, Jalil EM, Cardoso SW, Moreira RI, Calvet GA (2023). Mortality rates by gender and sexual orientation reveal a disproportionally high mortality among cisgender men of unknown sexual orientation and men who have sex with women in a cohort of people living with HIV in Rio De Janeiro, Brazil. Brazilian J Infect Dis.

[39] Jackson SS, Brown J, Pfeiffer RM, Shrewsbury D, O’Callaghan S, Berner AM (2023). Analysis of mortality among transgender and gender diverse adults in England. JAMA Netw Open.

[40] Crimmins EM, Shim H, Zhang YS, Kim JK (2019). Differences between Men and Women in Mortality and the Health dimensions of the morbidity process. Clin Chem.

[41] Lenart P, Kuruczova D, Joshi PK, Bienertová-Vašků J (2019). Male mortality rates mirror mortality rates of older females. Sci Rep.

[42] Mota M, Santana AD, da Silva S, de Melo LR. Clara, esta sou eu! Nome, acesso à saúde e sofrimento social entre pessoas transgênero. Interface - Comunicação, Saúde, Educação. 2022;26.

[43] Pinto IV, Andrade SS, de Rodrigues A, Santos LL, Marinho MAS, Benício MMA (2020). Perfil das notificações de violências em lésbicas, gays, bissexuais, travestis e transexuais registradas no Sistema De Informação De Agravos De Notificação, Brasil, 2015 a 2017. Revista Brasileira De Epidemiologia.

[44] Flanagin A, Frey T, Christiansen SL, Bauchner H (2021). The reporting of race and ethnicity in Medical and Science Journals. JAMA.

[45] Santos RV, Bastos JL, Kaingang JD, Batista LE. Cabem recomendações para usos de raça nas publicações em saúde? Um enfático sim, inclusive pelas implicações para as práticas antirracistas. Cad Saude Publica. 2022;38. 10.1590/0102-311X00021922 35293436

[46] Correia LO dos, Padilha S, Vasconcelos BM. Métodos para avaliar a completitude dos Dados Dos Sistemas De informação em saúde do Brasil: uma revisão sistemática. Cien Saude Colet. 2014;19:4467–78. 10.1590/1413-812320141911.02822013 25351313

[47] Cobo B, Cruz C, Dick PC (2021). Desigualdades De gênero E raciais no acesso e uso dos serviços De atenção primária à saúde no Brasil. Cien Saude Colet.

[48] Evedove AUD, Dellaroza MS, Carvalho WO, Loch MR (2021). Mudança na situação conjugal e incidência de comportamentos de proteção à saúde em adultos com 40 anos ou mais: Estudo VigiCardio (2011–2015). Cad Saude Colet.

[49] Malta DC, Bernal RTI, Lima MG, Silva AG da, Szwarcwald CL, de Barros MB. A. Socioeconomic inequalities related to noncommunicable diseases and their limitations: National Health Survey, 2019. Revista Brasileira de Epidemiologia. 2021;24 suppl 2. 10.1590/1980-549720210011.supl.2 34910065

[50] Castro CMS, Costa MFL, Cesar CC, Neves JAB, Sampaio RF (2019). Influência Da Escolaridade E das condições de saúde no trabalho remunerado de idosos brasileiros. Cien Saude Colet.

[51] Alcântara DC, Caravaca-Morera JA, Peixoto EM, Rafael RDMR, De Andrade MDC, Gil AC (2022). Intersectionality and transsexuality in the process of discrimination: an integrative review. Revista Enfermagem UERJ.

[52] Leite FMC, Santos DF, Ribeiro LA, Tavares FL, Correa ES, Ribeiro LEP et al. Análise dos casos de violência interpessoal contra mulheres. Acta Paulista De Enfermagem. 2023;36.

[53] Lima VMda, Stochero F, Azeredo L, Moraes CM, de Hasselmann CL, Marques MH. ES. Characterization and completeness of notification sheet of violence against the older adults in Niterói-RJ, 2011–2020. Epidemiologia e Serviços de Saúde. 2023;32. 10.1590/S2237-96222023000100024 PMC10027045 36946831

[54] da Silva MMA, Mascarenhas MDM, Lima CM, Malta DC, Monteiro RA, de Freitas MG et al. Perfil do Inquérito de Violências e Acidentes em Serviços Sentinela de Urgência e Emergência. Epidemiologia e Serviços de Saúde. 2017;26:183–94. 10.5123/S1679-49742017000100019 28226020

[55] Rafael R, de MR, Jalil EM, Velasque L, de Friedman S, Ramos RK, Cunha M. CB, Intimate Partner Violence among Brazilian trans and Cisgender Women living with HIV or at HIV Risk during COVID-19 era: another epidemic? Transgend Health. 2023. 10.1089/trgh.2023.0057. 10.1089/trgh.2023.0057 PMC11937771 40151174

[56] Krug EG, Dahlberg LL, Mercy JA, Zwi AB, Lozano R. World report on violence and health. Geneva; 2002.

[57] Butler J (2004). Precarious life: the Powers of mourning and violence.

[58] Canavese D, Polidoro M, Signorelli MC, Moretti-Pires RO, Parker R, Terto (2022). V. A call for the urgent and definitive inclusion of gender identity and sexual orientation data in the Brazilian health information systems: what can we learn from the monkeypox outbreak?. Cien Saude Colet.

